# Estimating the proportions and latencies of reaction time outliers: A pooling method and case study of lexical decision tasks

**DOI:** 10.3758/s13428-024-02419-y

**Published:** 2024-05-29

**Authors:** Jeff Miller

**Affiliations:** Department of Psychology, University of Otago, PO Box 56, Dunedin, 9054 New Zealand

**Keywords:** Reaction time distributions, Outliers, Lexical decision task

## Abstract

A methodological problem in most reaction time (RT) studies is that some measured RTs may be outliers—that is, they may be very fast or very slow for reasons unconnected to the task-related processing of interest. Numerous ad hoc methods have been suggested to discriminate between such outliers and the valid RTs of interest, but it is extremely difficult to determine how well these methods work in practice because virtually nothing is known about the actual characteristics of outliers in real RT datasets. This article proposes a new method of pooling cumulative distribution function values for examining empirical RT distributions to assess both the proportions of outliers and their latencies relative to those of the valid RTs. As the method is developed, its strengths and weaknesses are examined using simulations based on previously suggested ad hoc models for RT outliers with particular assumed proportions and distributions of valid RTs and outliers. The method is then applied to several large RT datasets from lexical decision tasks, and the results provide the first empirically based description of outlier RTs. For these datasets, fewer than 1% of the RTs seem to be outliers, and the median outlier latency appears to be approximately 4–6 standard deviations of RT above the mean of the valid RT distribution.

Since the earliest days of experimental psychology, reaction time (RT) has been one of the most common measures of mental processes (Boring, 1929; Brebner & Welford, 1980). This measure is objective, is available cheaply and unobtrusively in a wide variety of tasks, and bears an obvious relation to the mental processes being measured. It also provides a convenient foundation for detailed quantitative models of the processes underlying specific tasks (Luce, 1986).

At the same time, RT presents challenges to researchers because of its well-documented large variability. Substantially different RTs will be obtained in different trials with seemingly identical conditions—that is, trials from a single participant, tested in the same task, with the same stimulus and response. The distributions of such within-condition RTs typically have standard deviations that are approximately 10% or more of their means and are generally skewed, with long tails of slow RTs often extending out past 2–3 times the mean (Luce, 1986). RT researchers have become increasingly interested in exploring the complex characteristics of RT distributions because these can provide useful information beyond what is available in simple summary measures like means and medians (e.g., Balota & Yap, 2011; Heathcote, Popiel, & Mewhort, 1991; Miller, 1982; Ruthruff, 1996).

A practical problem in the study of RT distributions is that some of the more extreme RTs may actually be outliers that do not reflect relevant task processing. Although most observed RTs are presumably valid ones determined by the normal processes involved in performing the required task, a few of the observed RTs may be generated by anomalous processes in which the researcher is not interested. For example, RTs may be too short (i.e., “fast outliers”) if participants accidentally trigger the response apparatus before performing the task or if they simply anticipate the stimulus in an attempt to perform quickly (Ollman, 1966). Likewise, measured RTs may be inappropriately long (i.e., “slow outliers”) if participants are momentarily distracted from the task (e.g., by coughing, fatigue, extraneous thoughts) or if a mechanical response does not register and has to be repeated because of some motor glitch. Naturally, to obtain a more realistic picture of the true distributions of processing times, RT researchers should identify and exclude such outlier RTs.

In most tasks, fast outliers are relatively easy to recognize. There is general agreement that most participants have a physiological lower limit of RT on the order of 100–150 ms (e.g., Luce, 1986), so RTs less than this can be excluded as fast outliers on theoretical grounds. Even in choice RT tasks where this physiological lower limit is not approached, the lower tails of empirical RT distributions often fall off sharply and accuracy is at chance for the fastest RTs, making it relatively easy to detect fast outliers that are distinct from the rest of the distribution (Cousineau et al., 2023).

Slow outliers are more difficult to identify, because they can normally only be detected when they are unrealistically large relative to the valid RTs. Yet, most stochastic latency mechanisms predict that quite long valid RTs will occasionally be generated by genuine task-related processes (e.g., Luce, 1986), which makes it very difficult to say just how large is “unrealistically large.” For example, with the long tails commonly found at the high end of skewed theoretical RT distributions, the slowest valid RT in a given set of trials (i.e., for a given participant in a given condition) could be several hundred milliseconds slower than the second-slowest valid RT. This makes it very difficult to distinguish between those RTs that are part of the real distribution (i.e., valid RTs) and those that are slow outliers. Obviously, researchers studying task-related processing mechanisms should retain slow valid RTs, because these provide potentially informative clues about those mechanisms. Thus, RTs should not be excluded as outliers simply because they are slow, but only when they are so slow that they were unlikely to have been valid RTs. The question is, just how slow is that? Unfortunately, no empirically-based description of these outliers has yet been developed (Berger & Kiefer, 2021; Ulrich & Miller, 1994), and it is difficult to develop methods for discriminating between the valid RTs and the slow outliers without knowing anything about how the outliers are distributed.

The purpose of this paper was to investigate the empirical characteristics of outliers in typical RT tasks. Since outliers are expected to be rare, very large datasets are needed to get an accurate picture of them. Fortunately, as will be described later, four large lexical decision task mega-studies have been conducted that can be used for this purpose. Using these datasets, a new method of pooling cumulative distribution function (CDF) values was developed in order to obtain information about both the proportions of outliers present in the empirical datasets and about the overall latencies of those outliers. It should be emphasized that the immediate goal of this work was *not* to obtain an improved method of classifying individual RTs as valid versus outliers. Instead, the method provides information about the characteristics of outliers *in aggregate*, somewhat analogous to techniques for estimating the proportions of people who would answer “yes” to sensitive questions without obtaining a definitive answer for any particular individual (e.g., asking elite athletes “Have you knowingly violated anti-doping regulations by using a prohibited substance or method in the past 12 months?”; Ulrich et al., 2018). Aggregate information about the proportions and latencies of outliers has implications regarding the effects of outliers on various methods of RT analysis and the utility of current methods of outlier exclusion. Eventually, it should also contribute to the development of improved methods of excluding individual RTs as outliers.

The organization of this paper is as follows. The next section describes several ad hoc models of outliers that have been suggested—with no specific empirical basis—in previous studies examining the efficiency of various RT data analysis procedures (e.g., procedures for outlier exclusion). These models are useful test beds in which to evaluate the proposed method for investigating outliers, because in their cases the truth about outliers is known a priori from the models’ assumptions. Following that, I propose a new method of CDF pooling across multiple participants to obtain information about the distributions of valid RTs and outliers—especially high-resolution information about the distribution tails where the outliers are most likely to be discernible. In the subsequent sections, I apply the new method both to RTs simulated from the previously suggested ad hoc outlier models, with known probabilities and distributions of valid and slow outlier RTs, and to real RTs from lexical decision tasks. Applying the method to simulated RTs helps to illustrate the method and more importantly to investigate its accuracy in uncovering the known characteristics of the simulated outliers. These analyses show that the pooling method can provide specific insights about the outliers present in a dataset. I then apply the method to real RTs from the lexical decision mega-studies to develop a picture of the outliers that can be expected in this important type of RT experiment. To preview the results, it appears that slow outliers are rare—less than 1% of trials—and that their latencies overlap with approximately the top 1–1.5% of the distribution of valid RTs.

## Previous ad hoc models of outliers

Various intuitively appealing procedures for identifying outliers have been proposed and are often used (e.g., Berger & Kiefer, 2021; Cousineau & Chartier, 2010). The performance of these procedures has generally been assessed within the context of simulated datasets, assuming specific proportions of outliers and distributions of their latencies (see Miller, 2023, for an exception). Given that no empirically based description of RT outliers has yet been developed (Berger & Kiefer, 2021; Ulrich & Miller, 1994), these assumptions have been somewhat arbitrary. Moreover, the divergence among the assumed outlier distributions illustrates the lack of consensus about the characteristics of these distributions.

**Fig. 1:**
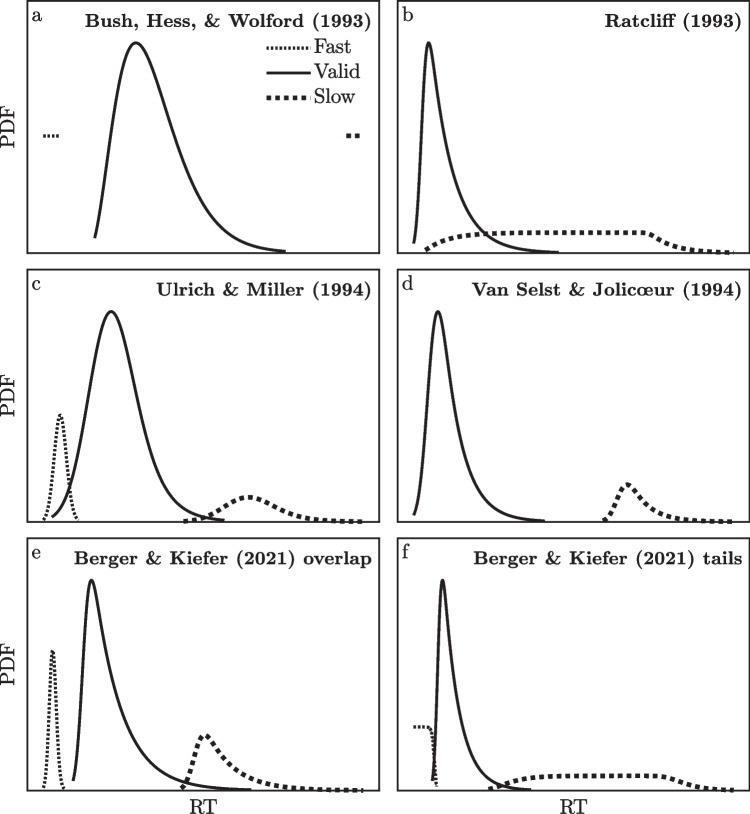
Examples of probability density functions (PDFs) of valid reaction times (RTs), fast outliers, and slow outliers assumed by different researchers performing simulations examining RT analysis methods. The total areas of the different outlier distributions have been scaled upwards to facilitate visual comparisons among the different assumptions. **a** Gamma distribution of valid RTs. Fast outliers (2%) are 3 SDs below the mean valid RT and slow outliers (1%) are 6 SDs above that mean. **b** Ex-Gaussian distribution of valid RTs with slow outliers formed by adding a uniform 0–2000 random number to a valid RT. **c** Ex-Gaussian distributions of valid RTs, fast outliers, and slow outliers. **d** Ex-Gaussian distributions of valid RTs and slow outliers. **e** Ex-Gaussian distributions of valid RTs, fast outliers, and slow outliers. **f** Ex-Gaussian distribution of valid RTs. Fast outliers are uniformly distributed between 100 ms and the fastest valid RT in the sample. Slow outliers are slower than the slowest valid RT in the sample by an amount that varies uniformly from 0–2000 ms. Distributions of the fastest and slowest RTs in the samples were computed for a sample size of 60 trials

Figure 1 illustrates representative examples of some previously assumed outlier distributions. The figure shows examples of hypothetical valid and outlier RT distributions for a single participant in a single condition; some simulation studies have compared different analysis options with a single set of fixed distributions like these, whereas other studies have allowed participant-to-participant variation in the parameters of the valid and outlier distributions.

For example, Bush et al. (1993; henceforth, BHW) carried out a simulation with valid RTs coming from a chi-square distribution, plus 2% fast outliers and 1% slow outliers. Their outliers were constant values three standard deviations below the valid RT mean or six standard deviations above it^1^. In contrast, other researchers have generally assumed that valid RTs come from the ex-Gaussian distribution, which has parameters *µ*, *σ*, and *τ* (e.g., Balota & Yap, 2011; Hohle, 1965; Luce, 1986), but they have assumed different distributions for outliers. For example, in some simulations Ratcliff (1993; henceforth, RAT) included 10% slow outliers that were modelled as regular RTs with an added start-up delay uniformly distributed from 0–2000 ms (see also, Vankov, 2023). As can be seen in Fig. 1b, this produces a very wide range of slow outliers and a substantial overlap of the distributions of valid RTs and slow outliers. Slow outliers can be nearly as fast as the fastest valid RTs (i.e., fast valid RTs with a near-zero start-up delay), but they can also be 2 s slower than the slowest valid RTs (i.e., slow valid RTs with a nearly 2 s delay)—quite different from the outlier distributions assumed by Bush et al. (1993). In various conditions, Ulrich and Miller (1994; henceforth, UM) and Van Selst and Jolicœur (1994; henceforth, VSJ) adopted specific ex-Gaussian distributions for the fast and slow outliers, with smaller or larger differences between the distributions of valid RTs and outliers. Berger and Kiefer (2021) generated valid RTs as ex-Gaussians whose parameters varied randomly across simulated participants, and they generated outliers using one of two distinct and somewhat complicated methods known as “overlap” and “tails” (henceforth, BK_overlap_ and BK_tails_; see Appendix 1 for details)^2^.

It is now well established by simulation studies that the effectiveness of different RT analysis procedures depends—among other things—on the frequencies and distributions of the simulated outliers (e.g., Berger & Kiefer, 2021; Bush et al., 1993; Ulrich & Miller, 1994). Thus, information about real outlier frequencies and latencies would clearly be very useful for deciding how researchers can most effectively analyze their real RT data. The next section describes a new method developed to obtain such information.

## Pooling CDF values

In order to get a fine-grained picture of an RT distribution—and especially of its relatively rare outlier RTs—it is essential to have a frequency distribution tabulated across many thousands of observed trials. This presents a practical problem, because few participants are willing to provide that many trials per condition and—even if they did—there might be day-to-day variation due to practice or other factors. In order to get enough trials to study outliers, it would be helpful to have a method of pooling RTs from different distributions (i.e., from multiple participants and/or sessions) into a single overall distribution. As is described in detail in this section, the method proposed here is based on pooling estimated CDF values of the RTs from different distributions. Recall that the CDF value of a given score is the proportion of scores in its distribution that are smaller. For example, for the value of *X* = 1*.*96 from the standard normal distribution, the CDF value is *Y* = *F*(1*.*96) = 0*.*975 because 97.5% of this distribution lies below the value of 1.96.

The rationale for the present CDF pooling is based on the following statistical fact that is central to the current approach: For any continuous random variable *X*, the distribution of the CDF values *Y* = *F*(*X*) is uniform over the interval 0–1, where *F* is the cumulative distribution function of *X*. Intuitively, this simply means that any score *X* is equally likely to come from every percentile of its distribution, from the lowest 1% [i.e., 0 *< F*(*X*) *≤* 0*.*01], up to the highest 1% [0*.*99 *< F*(*X*) *≤* 1].

**Fig. 2:**
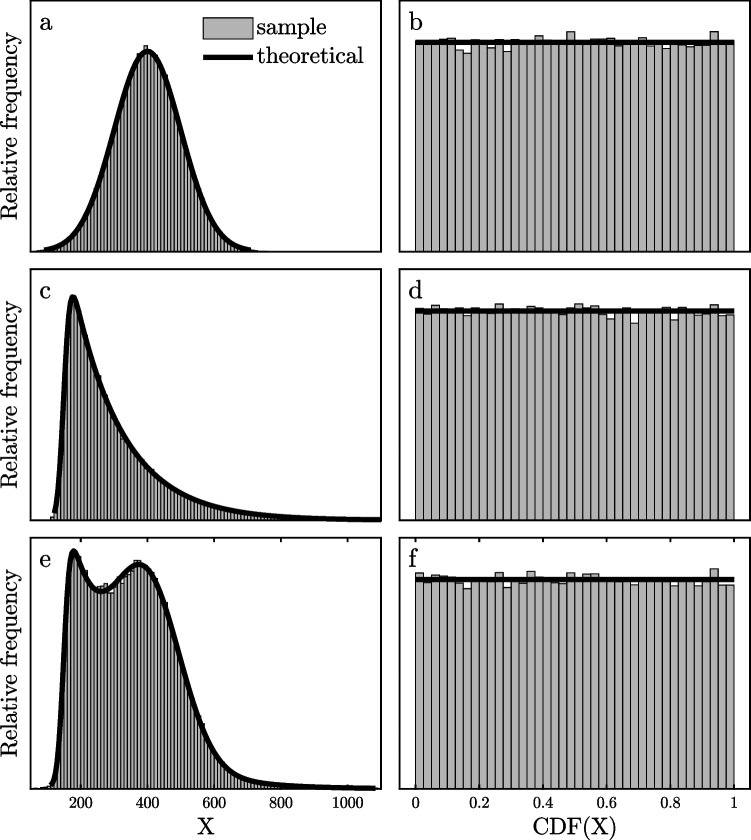
Theoretical probability density functions (lines) and frequency histograms of simulated observations (bars) with the PDFs scaled to cover the same areas as the histograms. **a** A theoretical normal distribution and a histogram of simulated values from it. **b** The theoretical distribution of the cumulative distribution function (CDF) values of scores from the normal distribution in **a**, and a histogram of the CDF values of the simulated observations in **a**. **c** A theoretical ex-Gaussian distribution and a histogram of simulated values from it. **d** The theoretical distribution of the CDF values of scores from that ex-Gaussian distribution and a histogram of the CDF values of the simulated observations in **c**. **e** The theoretical distribution of values pooled across the distributions in **a** and **c**, and the histogram of pooled simulated values. **f** The theoretical distribution of the pooled CDF values from **b** and **d** and the histogram of pooled simulated values

Figure 2 illustrates this fact. In Fig. 2a, the solid line shows a normal distribution with *µ* = 400 and *σ* = 100 superimposed over a histogram of 100,000 random *X* values sampled from this distribution. To construct the histogram in Fig. 2b, the CDF value *Y* = *F*(*X*) of each random *X* value was computed relative to that true underlying normal distribution with its known *µ* and *σ*. The resulting histogram of these *Y* values is approximately uniform, and there are slight deviations from uniformity—just as there are slight deviations from the normal curve in Fig. 2a—only because the sample size was finite. Because the *X* values in the tails of the distribution are less likely than those in the center (Fig. 2a), the 1% ranges of *X* values must be correspondingly wider in the tails than in the center so that it will be equally likely (i.e., 1%) to get a score in any given 1% range of CDF values, by definition.

Figure 2c and d show a parallel example starting with an ex-Gaussian distribution, which is a distribution typically found to provide a good approximation of empirical RTs (e.g., Balota & Yap, 2011; Hohle, 1965; Luce, 1986). Although the underlying ex-Gaussian is skewed (Fig. 2c), the distribution of the CDF values computed for observations from that distribution is again uniform.

The fact that CDF values are always uniformly distributed from 0–1 means that the CDF values of scores from different distributions can be tabulated within a single combined distribution (i.e., “pooled”) without changing the shape of the pooled CDF distribution, even though the shapes of the original distributions are not preserved if the raw values from the original distributions are pooled. For example, Fig. 2e shows the pooled frequency distribution tabulated across all 200,000 scores from the normal and ex-Gaussian distributions shown in Fig. 2a and c. This pooled distribution looks very different from either of the underlying distributions from which it was obtained—it is obviously neither normal nor ex-Gaussian. Nonetheless, as shown in Fig. 2f, the 200,000 pooled CDF values of the scores from these different distributions show the expected uniform distribution over the 0–1 interval. This must be the case, because the CDF values of the *X* values of the scores in different underlying distributions—relative to their own distributions—necessarily have the same uniform distribution even though the distributions of the underlying *X* values differ.

**Fig. 3:**
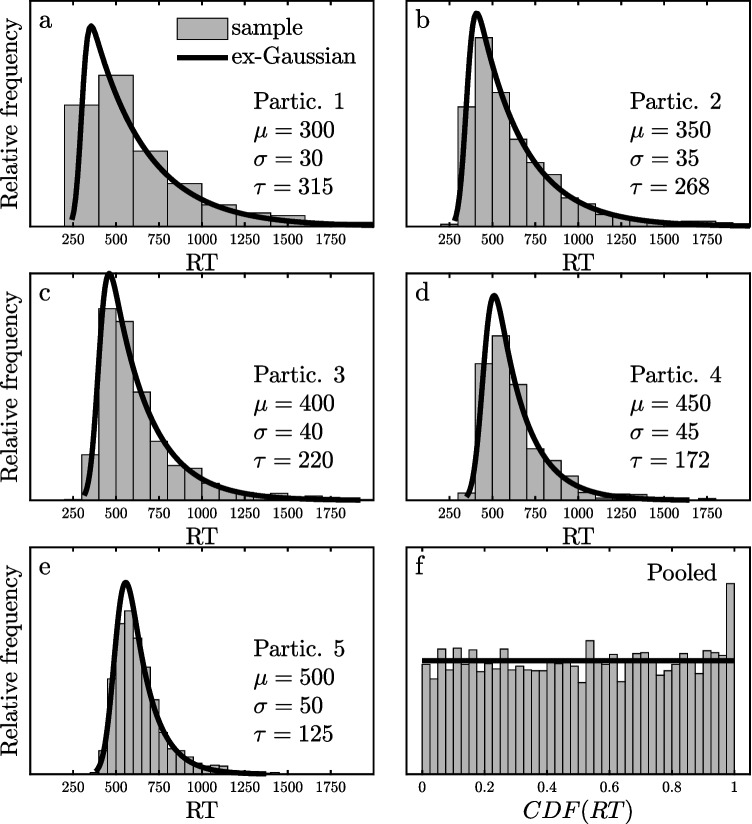
Histograms (bars) and associated theoretical probability density functions (lines) scaled to cover the same areas as the histograms. **a**–**e** Histograms of 1000 randomly sampled reaction times (RTs) for each of five hypothetical participants (Participants 1–5). For each participant, 985 valid RTs were sampled from an ex-Gaussian distribution with the parameters indicated, and 15 slow outliers were sampled from the top 1% of that distribution. **f** Pooled frequency distribution of the CDF values of all participants’ RTs scored within their individual ex-Gaussian valid RT distributions (bin size = 2.5%)

The presence of outliers would perturb the expected uniform 0–1 CDF distribution, however, if the CDF values of the outliers were computed relative to the valid distribution. By definition, fast and slow outliers would be concentrated, respectively, in the lowest and highest 1% or so of the valid distribution, so their CDFs would all be near zero or 1. To the extent that fast and slow outliers were present in the dataset, they would tend to increase the frequency of near-zero and near-1 CDFs, which would produce increased frequency “spikes” in the lowest and highest bins of a CDF histogram. Although this increase might not be visible in a single participant’s frequency distribution because of the limited number of trials available, it could well become clear if CDF values were pooled across participants. Figure 3 illustrates this in a simulated dataset consisting of five participants with various true ex-Gaussian distributions of valid RTs, depicted by the solid lines in Fig. 3a–e. The *µ*, *σ*, and *τ* of each participant’s valid RT distribution are indicated in the figure, and 985 RTs were randomly selected from this distribution for each participant. To model the presence of 1.5% slow outliers, 15 additional RTs were sampled specifically from the top 1% of the participant’s true distribution. The histograms of the 1000 RTs from each participant are shown in Fig. 3a–e, and it is impossible to see a clear indication of outliers in these histograms. This illustrates the difficulty of identifying outliers in the long slow tails of observed RT distributions, which was mentioned earlier. The CDFs of each participant’s 1000 RTs were then computed relative to *that participant’s true distribution of valid RTs*. The histogram of these 5000 pooled CDF values is shown in Fig. 3f. The presence of the simulated slow outliers is clearly visible in the spike for the highest CDF bin, reflecting the fact that the 15 outlier RTs for each participant were all selected from that bin in their participant’s own valid RT distribution. Thus, pooling of CDF values across participants may provide a view of the outliers that cannot be seen by looking at the participants’ individual distributions of observed RTs.

## Evaluation of pooling method in theory

To see how well the CDF pooling method can reveal the presence of various types of RT outliers, I first checked its performance on simulated datasets with known outliers and known distributions of valid RTs. This is a best-case scenario for the method in the sense that all CDFs can be computed relative to known true valid RT distributions. None of these computations could be carried out with real data, however, because in that case the true underlying distributions are unknown. Thus, the subsequent section evaluates how well the method would work in practice, where the unknown valid RT distributions must be estimated from the observed RTs, which could themselves include outliers.

The theoretical outlier models depicted in Fig. 1 were used for this purpose, and separate simulations were conducted with each model using relatively low, medium, and high proportions of outliers. For each combination of model and outlier proportion, 200 RTs were randomly generated for each of 1000 simulated participants. For all models, the valid RTs were randomly generated from ex-Gaussian distributions to simulate trial-to-trial variability. To simulate participant-to-participant variability, the values of each simulated participant’s valid RT ex-Gaussian parameters *µ*, *σ*, and *τ* were randomly chosen from separate hyper-parameter distributions suggested by Berger and Kiefer (2021). Each participant’s true proportion of slow outliers was also chosen randomly, and in different simulations these proportions were chosen from distributions with relatively low, medium, or high proportions of outliers overall (i.e., mean proportions of 1%, 2.5%, or 5%). The numerical values of the outlier RTs were generated differently for each outlier model in accordance with the assumptions of that model (Fig. 1; see Appendix 1 for additional details). After the simulated 200 RTs were randomly generated for each participant, the CDFs of all RTs—including any outliers—were computed relative to that participant’s valid RT distribution with its true values of *µ*, *σ*, and *τ*, and these CDF values were pooled across all simulated participants. Since all of the slow outlier CDF values would be near 1 within the valid RT distributions, they should produce a spike at the high end of each CDF distribution.

**Fig. 4:**
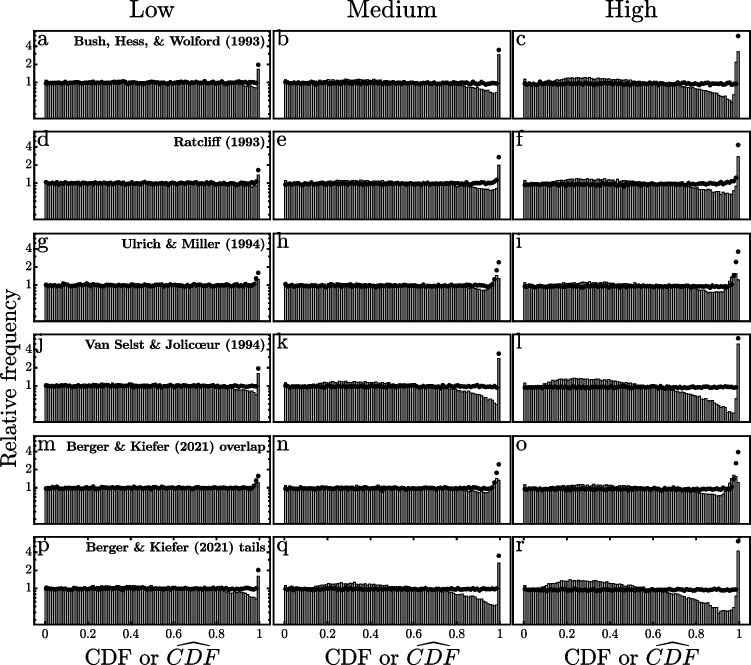
Relative frequencies of the cumulative distribution function (CDF and $\widehat{{\text{CDF}}}$) values of reaction times (RTs) simulated from the outlier models depicted in Fig. 1. The dots show the frequencies of CDF values computed from each simulated participant’s true underlying ex-Gaussian RT distribution. The histograms show the frequencies of $\widehat{{\text{CDF}}}$ values computed from ex-Gaussian distributions with parameters estimated from each participant’s simulated RTs and with the proportion of outliers randomly selected for each participant from uniform distributions ranging from zero to 0.02, 0.05, or 0.1 in simulations with low, medium, and high proportions of outliers, respectively

The black dots in Fig. 4 summarize the most important results of these simulations. These dots represent the tops of histograms showing the frequencies of the different CDF values pooled across simulated participants for each model and each outlier proportion, and these show the expected pattern. CDF values are almost uniformly distributed across most of the possible 0–1 range, with only small fluctuations due to the limited sample of 1000 *×* 200 = 200*,*000 trials. In contrast, there is clearly a frequency spike indicated by the rising black dots in the largest CDF bin due to the presence of slow outliers. Furthermore, this spike is larger when the proportion of outliers is higher. Thus, these frequencies support the idea that the CDF pooling method can provide a window on RT outliers. Small differences among models can also be seen in the second- and third-highest bins, with elevated frequencies in these bins mainly for models allowing some overlap of the valid and outlier RT distributions (cf. Fig. 1), suggesting that empirical data might even provide evidence concerning the amount of overlap between the distributions of valid and outlier RTs, as will be considered later.

## Evaluation of pooling method with estimated parameter values

Unfortunately, in actual practice with real datasets, researchers cannot tabulate the frequencies of CDF values analogous to those shown by the black dots in Fig. 4. Even under the assumption that every participant’s valid RTs come from an ex-Gaussian distribution, the true values of each participant’s underlying *µ*, *σ*, and *τ* parameters are needed in order to compute these CDF values, but with real participants the true values of these parameters are unknown. At best, the researcher can only *estimate* the values of these parameters from the participant’s observed RTs, and then compute an *estimated* CDF, $\widehat{CDF}$, of each observed RT relative to the ex-Gaussian distribution with the estimated parameter values^3^. The problem of unknown parameter values is of course ubiquitous in model fitting, since it is always necessary to estimate the true values of hypothesized parameters from observed data. For the present CDF pooling method, parameter estimation seems problematic not only because the *µ*, *σ*, and *τ* estimates obtained from a sample of RTs will deviate from the true values due to sampling but also—and more seriously—because the outliers within an RT sample can distort the estimates of the valid RT distribution’s parameters from that sample.

Fortunately, it is possible to see how seriously the CDF pooling method would be compromised by the use of estimated rather than true *µ*, *σ*, and *τ* values. For each of the participants simulated in creating Fig. 4, the 200 simulated RTs were used to compute maximum likelihood estimates of $\widehat{\mu }$, $\widehat{\sigma }$, and $\widehat{\tau }$, just as a researcher could do with real observed RTs (e.g., Lacouture & Cousineau, 2008). The estimated $\widehat{CDF}$ of each simulated RT from each participant was then computed from the ex-Gaussian distribution with the estimated values $\widehat{\mu }$, $\widehat{\sigma }$, and $\widehat{\tau }$. The gray histograms in Fig. 4 show the relative frequencies of these 200,000 $\widehat{CDF}$ values pooled across simulated participants for each model and proportion of outliers.

The effects of the previously mentioned distortions of the pooled $\widehat{CDF}$ values due to estimation of the ex-Gaussian parameters can be seen as the difference between the black dots (CDFs) and the gray histogram bars ($\widehat{CDF}$ s) shown in Fig. 4. There is very little distortion in the simulations with low probabilities of outliers, as indicated by nearly uniform $\widehat{CDF}$ frequencies across most of the 0–1 range, with a spike in the highest $\widehat{CDF}$ bin, similar to the pattern with CDFs. The distortion increases as the outlier probability increases, with clear departures from a uniform $\widehat{CDF}$ distribution, especially for $\widehat{CDF}$ values greater than 0.8 for some of the outlier models (e.g., VSJ and BK_tails_). In most cases it is still possible to see an increased frequency of $\widehat{CDF}$ values in the highest bin, however, suggesting again that the pooling method may be able to reveal the presence of outliers using estimated rather than true CDF values and thus that it might be informative with real data.

The high-end spikes shown in Fig. 4 are consistently smaller for $\widehat{CDF}$ values computed from estimated parameters than for CDFs computed from true parameters, especially when the proportion of outliers is high. This must be kept in mind when applying the method to real RT datasets, because it suggests that the method is likely to underestimate the true proportion of outliers. This is to be expected, because the presence of slow outliers in the RT samples biases the estimates of the ex-Gaussian parameters—particularly *τ* (see Table 5)—and thereby distorts the estimated $\widehat{CDF}$ values relative to the true CDFs. Fortunately, the distortion appears relatively small when the proportions of outliers are low or medium, as would be expected in most carefully conducted RT studies.

In summary, the main message of Fig. 4 is that slow outliers can generally be seen as high-end spikes in the distributions of $\widehat{CDF}$ values computed and pooled across participants in the proposed manner. Depending on the exact relationships between the valid and slow outlier distributions and the proportion of outliers, the spikes can be limited to the most extreme $\widehat{CDF}$ values or can extend down to slightly lower $\widehat{CDF}$ values. As expected, these spikes are larger when there is a larger proportion of outliers. Together, these simulation results indicate that the proposed pooling method can provide some information about the presence of outliers in real RT samples. The following two sections report results of applying this method to real data from four mega-studies of RTs in lexical decision tasks.

## The Semantic Priming Project dataset

Given the preceding simulation-based evidence that the CDF pooling method provides useful information about RT outliers, I next used the method to investigate outliers in real datasets. I used the data from mega-studies of the lexical decision task because this is a well-known and important RT task within experimental psychology and because these datasets are unusually large and thus provide rich information for characterizing the distributions of both valid RTs and occasional outliers. I first applied the method to the publicly available Semantic Priming Project (SPP) dataset of Hutchison et al. (2013), which includes approximately 1600 trial-by-trial RTs recorded from each of 503 participants categorizing individual letter strings as words versus nonwords in a lexical decision task. For completeness, this section provides a detailed description of the application of the method to this dataset. As a check on the generality of the conclusions from the SPP dataset, parallel investigations were also carried out for several other lexical decision task datasets for comparison purposes, and their results are reported in the following section.

Recorded RTs in the SPP dataset range from 0–2999 ms, with the upper limit reflecting the maximum interval allowed for responding before the trial ended. For the present analysis, RTs less than 150 ms (*≈* 0*.*5%) were excluded as unrealistically fast, because this value is less than that typically regarded as a lower estimate for simple RT and the present choice-task RTs should be even slower than simple RTs^4^. It may seem strange to eliminate obviously too-fast RTs from an analysis designed to identify outliers, but retaining them would increase noise and make it more difficult to assess the presence of outliers among the RTs whose validity is in doubt. Thus, the present method should be viewed as a way of investigating what proportion of the *questionable* RTs are outliers, after the obvious outliers have been removed. No RT upper bound was set because participants might conceivably need several seconds to decide whether a letter string is a word or not, and the maximum RT recording interval of 2999 ms eliminates the possibility of any obviously too-long RTs (e.g., 30 s).

Following the exclusion of the unrealistically fast RTs, a preliminary ANOVA on individual-participant mean correct RTs showed highly significant effects of word versus nonword trial, response repetition versus response alternation (i.e., whether the preceding trial’s response had been the same or different), and the interaction of these two factors (*p <* 0*.*001 and *η*_*p*_^2^ *>* 0*.*34). In view of the different mean RTs in these four conditions, it seemed appropriate to examine their RT distributions separately. Thus, the correct RTs of each participant were approximately equally divided across four conditions defined by whether the current stimulus was a word versus nonword and by response repetitions versus alternations, resulting in approximately 400 RTs per participant in each condition. The CDF pooling method was then applied separately and independently to the RTs in each of these four conditions, providing a type of within-experiment replication of the analysis.

As mentioned earlier, the ex-Gaussian distribution is typically used as a model for RT distributions. Given the crucial status of the assumed distribution model for the computation of $\widehat{CDF}$ values, however, it is important to confirm that this distribution is actually a good model for the 2012 RT distributions observed in the SPP study (i.e., 503 participants times four conditions). To that end, as described in detail in Appendix 2, a preliminary analysis was carried out to compare the fits of the observed RTs to the ex-Gaussian distribution versus their fits to 29 other candidate RT distributions. Each of 30 candidate RT distributions described in Appendix 2 was fit separately to each of 2012 observed RT distributions, and the ex-Gaussian emerged as the best-fitting distribution. Thus, for each of the 2012 combinations of participant and condition, ex-Gaussian parameter estimates $\widehat{\mu }$, $\widehat{\sigma }$, and $\widehat{\tau }$ were determined by maximum likelihood, and the $\widehat{CDF}$ of each individual RT was computed relative to the ex-Gaussian distribution with parameters equal to those estimates.

**Fig. 5:**
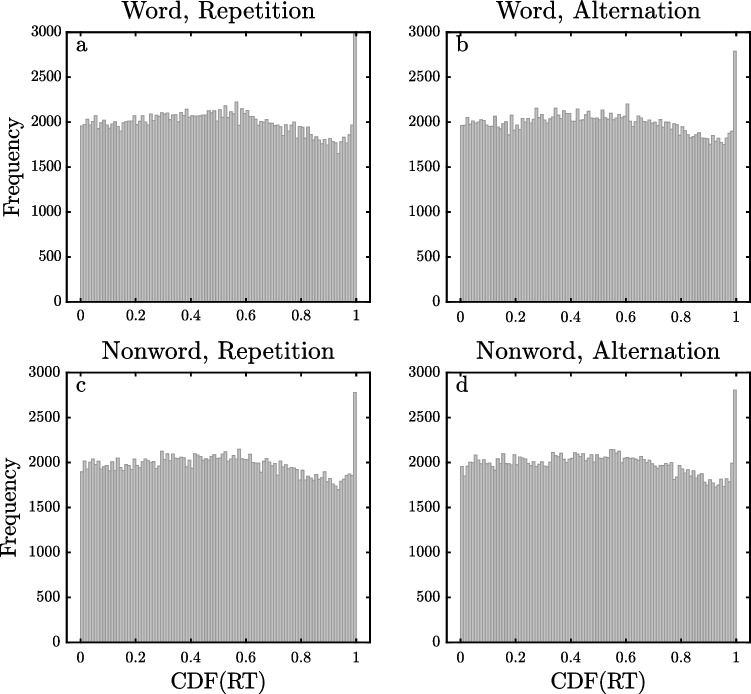
Histogram of pooled estimated cumulative distribution function ($\widehat{{\text{CDF}}}$) values of RTs for the Semantic Priming Project dataset

Figure 5 shows the pooled distributions of $\widehat{CDF}$ values for correct responses in each of four conditions from all participants in the SPP dataset (comprising a total of 796,066 individual RTs). Even though the four pooled distributions were computed from the RTs of separate sets of trials, the results are remarkably similar across conditions. In all four conditions, the distributions are fairly uniform over all but the highest bin, which has a much higher frequency than expected. For example, in their 0.99–1 bins, the four conditions contain 1.49%, 1.40%, 1.41%, and 1.41% of the $\widehat{CDF}$ values, and each of these percentages is significantly greater than the 1% that would be expected if there were no outliers (*p <* 0*.*00001 in each condition, by binomial tests). It seems plausible that the highest bins are over-represented in frequency precisely because they include occasional slow outliers. The lack of analogous spikes at the low ends of the four distributions suggests that fast outliers above the 150 ms cut-off were non-existent or extremely rare.

The fairly uniform distribution across the lower bins in Fig. 5 also provides further evidence that the ex-Gaussian is a reasonably good distributional model for the observed RTs. If each RT’s $\widehat{CDF}$ value was computed relative to a distribution substantially different from the true one—for example, a distribution from a different family or a distribution from the same family but with parameter values far from those estimated—then the pooled $\widehat{CDF}$ values would not generally be uniformly distributed. Thus, the reasonably uniform distributions of pooled $\widehat{CDF}$ values in Fig. 5—apart from the highest bin—provide reassurance both that the ex-Gaussian is a good distributional family for modelling the RTs in this task and that there were sufficient RTs per participant in each condition to get good estimates of the ex-Gaussian parameter values for each participant/condition combination. There is a small but consistent non-uniformity across the four frequency distributions in Fig. 5, however: there were slightly fewer RTs than expected with $\widehat{CDF}$ values in the range of 80–98%. Similar trends will also be seen in some of the datasets presented later, so this is probably not a chance finding. The explanation could well be the same as that for the analogous pattern seen in Fig. 4; the presence of slow outliers inflates the estimated values of *τ*, so there would not be quite as many RTs in the upper tail of the distribution as expected based on that inflated *τ*.

## Other lexical decision datasets

Analyses parallel to those carried out with the SPP dataset were also carried out with three other large lexical decision task datasets in order to check the robustness of the method and the generality of the conclusions. These datasets came from the Dutch Lexicon Project (DLP; Keuleers et al., 2010), the English Lexicon Project (ELP; Balota et al., 2007), and the French Lexicon Project (FLP; Ferrand et al., 2010). The DLP had extensive testing per person, with approximately 25,000 correct RTs from each of 39 participants, whereas the ELP and FLP datasets were more similar to the SPP dataset, including approximately 800–900 participants with 2000–3000 trials each. The maximum RT was 2000 ms in the DLP and FLP datasets due to a limited response interval, whereas it was 4000 ms in the ELP dataset. As with the SPP dataset, preliminary analyses of all datasets showed strong response repetition effects, so RT distributions were estimated for the same four conditions as the SPP dataset. For two of these three datasets, the ex-Gaussian provided the best fit to the individual participant and condition distributions, and for one it provided the second-best fit (Fig. B1), so it was again used for computation of the pooled $\widehat{CDF}$ values.

**Fig. 6:**
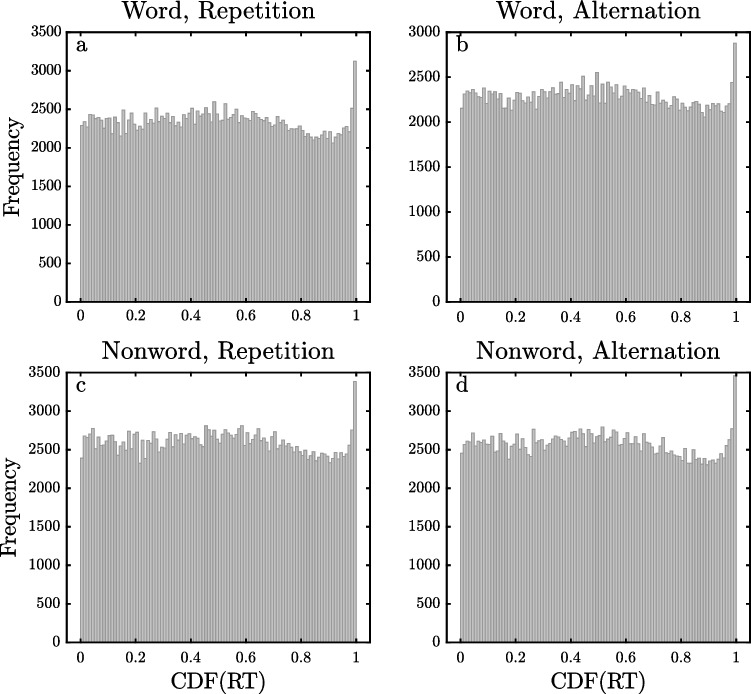
Histogram of pooled estimated cumulative distribution function ($\widehat{{\text{CDF}}}$) values of RTs for the Dutch Lexicon Project dataset

**Fig. 7:**
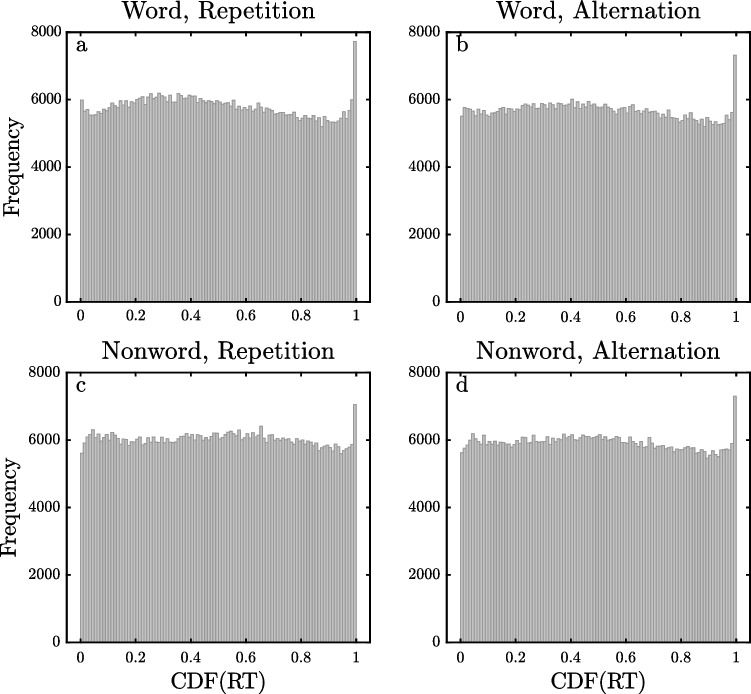
Histogram of pooled estimated cumulative distribution function ($\widehat{{\text{CDF}}}$) values of RTs for the English Lexicon Project dataset

**Fig. 8:**
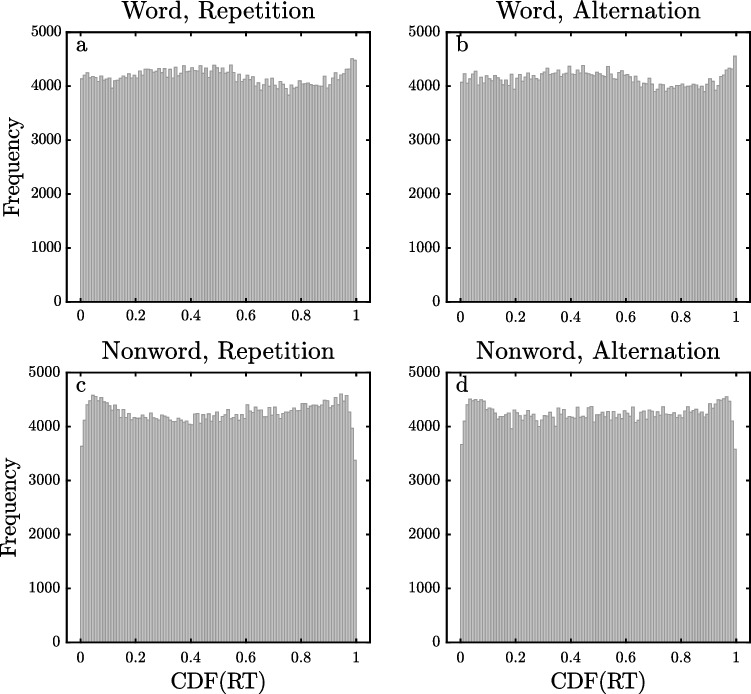
Histogram of pooled estimated cumulative distribution function ($\widehat{{\text{CDF}}}$) values of RTs for the French Lexicon Project dataset

Figures 6, 7 and 8 show the pooled $\widehat{CDF}$ distributions for the DLP, ELP, and FLP datasets, computed with the same method as the distributions for the SPP dataset shown in Fig. 5. The distributions for the DLP and ELP datasets are similar to those obtained with the SPP dataset. They show reasonably flat distributions of $\widehat{CDF}$ values across most of the 0–1 range except for spikes of increased frequency for $\widehat{CDF}$ values near 1, exactly as would be expected if the observed RTs came from ex-Gaussian distributions with occasional slow outliers. For the DLP and ELP datasets, the eight different 0.99–1 bins contain 1.17–1.35% of the trials, and all of these values are significantly greater than the 1% expected if there were no outliers (all *p <* 0*.*00001). For the FLP dataset, however, there are only small spikes at the highest $\widehat{CDF}$ values for the two word conditions (1.07% and 1.10%, both *p <* 0*.*001), with no such spikes in the distributions for nonwords. The weaker evidence for high outliers in the FLP dataset may be due to a procedural aspect of this study. As mentioned earlier, its maximum RT interval was only 2 s, so it may be that outliers were eliminated by trial termination. The maximum RT interval was also only 2 s in the DLP study, but the limitation on response time may have had less effect in this case because the participants had so much more practice with the task (i.e., approximately 25,000 versus 1800 trials per participant) and because the mean RT was correspondingly approximately 100 ms less.

## Estimating outlier proportions: Known outlier models

**Fig. 9:**
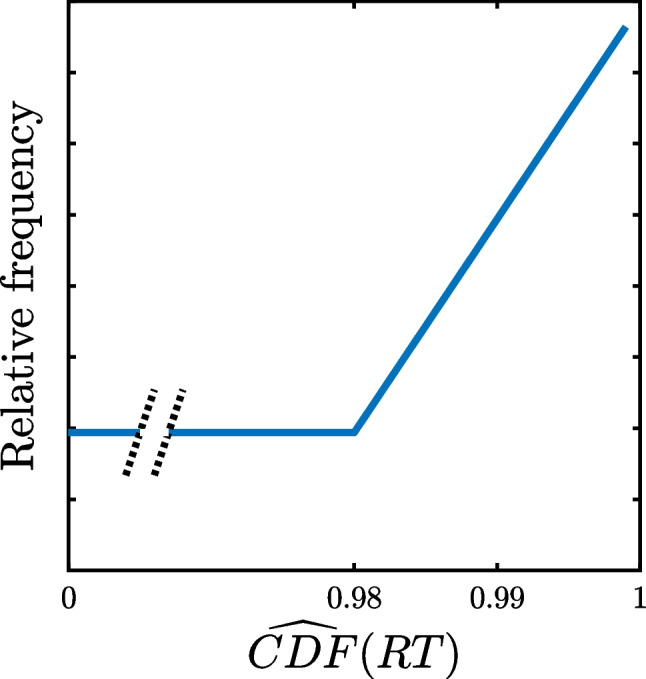
$\widehat{{\text{CDF}}}$ distribution predicted by the mixture model with p_o_ = 0.03 and ω = 0.98

If the $\widehat{CDF}$ distributions shown in Figs. 5, 6, 7, and 8 do reflect mixtures of the $\widehat{CDF}$ values of valid RTs and those of occasional slow outliers, then it should be possible to estimate, for each dataset, the proportion of outliers and their $\widehat{CDF}$ values with a simple mixture model. Let *p*_*o*_ be the probability of an outlier within the dataset under consideration. Then 1 *− p*_*o*_ is the proportion of valid RTs, and the $\widehat{CDF}$ values of these are approximately uniform 0–1, as already explained. The $\widehat{CDF}$ values for the slow outliers follow some other distribution whose exact shape is uncertain. Since the slow outliers are by definition quite large relative to valid RTs, this distribution should be concentrated near the maximum of the estimated valid distribution (i.e., the $\widehat{CDF}$ values of the outliers should be near 1.0), with decreasing frequency for smaller outlier $\widehat{CDF}$ values (e.g., 0.998, 0.997, and so on). Thus, it seems reasonable to model the $\widehat{CDF}$ values of the outliers as having a wedge-like half-triangular distribution with its mode at the maximum possible $\widehat{CDF}$ of 1.0 and a density function decreasing linearly to 0.0 at some minimum outlier $\widehat{CDF}$ , *ω*, which is a free parameter of the mixture model. For example, Fig. 9 shows an overall predicted $\widehat{CDF}$ distribution for this mixture model, combining $\widehat{CDF}$ values from valid RTs and outliers, with *p*_*o*_ = 0*.*03 and *ω* = 0*.*98. The distribution of $\widehat{CDF}$ values is perfectly flat for $\widehat{CDF}$ values from zero to *ω*, which can only come from valid RTs. It then increases linearly for $\widehat{CDF}$ values from *ω* to 1, which can come from both valid RTs and slow outliers, because of the increasing half-triangular density of outlier $\widehat{CDF}$ values. The linear increase that is evident for the largest $\widehat{CDF}$ values in Fig. 9 may not be visible in a pooled histogram of $\widehat{CDF}$ values, however, if *ω* falls within the histogram’s largest bin (e.g., with *ω* = 0*.*991 for the bin size of 0.01 shown in Fig. 4). In that case the top bin will show a frequency spike due to the outliers, but a narrower bin width would be needed to show the linear increase in frequencies of the different $\widehat{CDF}$ values within the highest bin.

In summary, the proposed mixture model has two free parameters: the outlier probability, *p*_*o*_, and the $\widehat{CDF}$ value (relative to the distribution of valid RTs) of the fastest possible slow outlier, *ω*. For both the simulated and real datasets explored previously, these parameters were estimated by maximum likelihood. The results of these fits for the histograms in Fig. 4 are summarized in Table 1. The parallel analyses for the real datasets (Figs. 5, 6, 7, and 8) are shown in Table 2.

**Table 1:** Estimated parameter values, p_o_ and ω, for fits of the mixture model to the distributions of pooled cumulative distribution function values computed from each simulated model with each proportion of slow outliers

Parameter and proportion of slow outliers						
	*p*_*o*_			*ω*		
Model	Low	Medium	High	Low	Medium	High
BHW	0.008	0.019	0.034	0.995	0.989	0.978
RAT	0.003	0.008	0.016	0.999	0.999	0.996
UM	0.005	0.011	0.019	0.971	0.955	0.935
VSJ	0.008	0.020	0.040	0.999	0.998	0.991
BK_overlap_	0.005	0.011	0.020	0.967	0.956	0.935
BK_tails_	0.006	0.014	0.027	1.000	1.000	0.998

**Table 2:** Estimated parameter values, p_o_ and ω, for fits of the mixture model to the distributions of pooled cumulative distribution function values computed from the lexical decision datasets shown in Figs. 5, 6, 7 and 8

Parameter, stimulus type, and repetition condition								
	${p}_{o}$				$\omega$			
	Word		Nonword		Word		Nonword	
Dataset	Rep.	Alt.	Rep.	Alt.	Rep.	Alt.	Rep.	Alt.
SPP	0.003	0.003	0.002	0.003	0.999	0.998	0.999	0.998
DLP	0.003	0.003	0.003	0.004	0.990	0.990	0.991	0.985
ELP	0.002	0.003	0.001	0.002	0.998	0.996	0.998	0.998
FLP	0.003	0.003	0.008	0.004	0.945	0.949	0.487	0.641

Rep. = repetition. Alt. = alternation

Since the true properties of the underlying distributions were known in the simulations using the models in Fig. 1, the parameter estimates in Table 1 can be evaluated relative to the known values of those parameters. For example, the true mean proportions of outliers were 0.01, 0.025, and 0.05 for the low, medium, and high conditions, respectively (see Appendix 1). The corresponding estimates of *p*_*o*_ appropriately increased with the true mean proportion of outliers for all models, suggesting that the model would be useful in comparing the proportions of outliers across different participant groups or conditions. Note, however, that *p*_*o*_ underestimates the true outlier probabilities in all cases, which must be kept in mind when interpreting obtained *p*_*o*_ values—the true values could be 1.5–2 times as large as the estimates. The estimate of *p*_*o*_ was most accurate with the BHW and VSJ models, and it was least accurate with the RAT and UM models. This suggests that the model’s estimate of *p*_*o*_ is especially likely to be too low when the distribution of outlier RTs overlaps with the distribution of valid RTs, as it does most strongly for the RAT and UM (see Fig. 1).

Across the six different outlier models, the estimates of *ω* clearly reflect the differing assumed degrees of overlap between the distributions of valid and outlier RTs. The estimates of *ω* are in the range of approximately 0.93–0.97 for the UM and BK_overlap_ models, correctly indicating that with these models the outlier distribution overlaps approximately 3–7% of the valid RT distribution at the slow end. For the BHW, VSJ, and BK_tails_ models, whose outliers are more extreme relative to the valid RTs, the estimates of *ω* were all greater than 0.99, correctly indicating that these outliers were larger than all but the very largest valid RTs. Thus, in these five cases the value of *ω* reflects outlier speeds fairly well.

Another clear trend in the *ω* estimates is that they decrease as the proportion of outliers increases. This is a consequence of the increasing bias in the estimates of *τ* for larger outlier proportions (see Table 5). As the valid distribution’s *τ* is increasingly overestimated with a larger outlier proportion, a given outlier RT will be located less far out in the tail of the fitted valid RT distribution and thus have a less extreme $\widehat{CDF}$ .

The *ω* estimates for the RAT model were badly incorrect, however. The estimates for this model were greater than 0.99 with all three outlier proportions, although the actual distribution of outlier RTs overlaps most of the valid RT distribution with this model (Fig. 1b). This misestimation of *ω* may be caused by the mixture model’s inability to identify the presence of many outliers of this type in the first place (i.e., small estimates of *p*_*o*_). Since the model does not recognize that outliers are present, it cannot provide good estimates of their $\widehat{CDF}$ values. It is difficult to see how any RT-based method could identify the proportion of outliers under this model in view of the extensive overlap of the distributions of valid and outlier RTs^5^.

In sum, the analyses of this section suggest that the pooled CDF approach and associated mixture model provide useful information about the proportions and relative latencies of outliers in RT data, especially when there is little overlap between the distributions of valid and outlier RTs, as could plausibly be expected in well-controlled experimental studies. Where there is only a small true proportion of outliers, the pooled $\widehat{CDF}$ values may be adequately described by a mixture model, and the true proportion of outliers seems to be approximately 1.5–2 times the size of the proportion estimated from this model. In addition, the *ω* parameter of the model gives a rough indication of the relative extremity of the outlier RTs within the distribution of valid RTs.

## Estimating outlier proportions: Lexical decision datasets

The mixture model developed in the previous section can be applied to the pooled $\widehat{CDF}$ values from the lexical decision tasks shown in Figs. 5, 6, 7, and 8 to estimate the proportions and latencies of outliers in these real datasets. The results obtained with simulated data in the previous section indicate that these estimates—although they will not be perfect—will provide useful approximations of the two mixture model parameters.

Table 2 shows the results of fitting of the mixture model to each of the real datasets of lexical decision RTs, and several points are noteworthy. First, the estimated outlier probabilities, *p*_*o*_, are low for all datasets and conditions. These estimated *p*_*o*_ values are slightly less than those obtained in simulations of the models of Fig. 1 with low (i.e., 0–2%) outlier probabilities, which suggests that there were probably fewer than 1% outliers overall in any of these datasets. Second, the large (i.e., nearly 1) *ω* values in the SPP, DLP, and ELP datasets suggest that these outliers fall within the top 1% of the valid RT distributions. Notably, the *ω* values are much lower for the FLP dataset, especially in the nonword conditions. Since there is little evidence of a slow outlier peak of $\widehat{CDF}$ values for this dataset in the first place (Fig. 8), it is not surprising that the mixture model is unable to provide stable estimates of the outliers’ locations within these $\widehat{CDF}$ distributions.

## Estimating the distribution of outlier RTs

The mixture model developed in the previous section provides an estimate of the distribution of $\widehat{CDF}$ values of the outlier RTs. It is possible to use this hypothesized distribution to estimate numerical outlier RT values in milliseconds relative to any researcher-selected reference distribution. Specifically, for an assumed reference distribution of valid RTs, one can work backwards from the estimated distribution of outlier $\widehat{CDF}$ values to compute the corresponding outlier RTs in milliseconds by “looking up” each of the outlier $\widehat{CDF}$ values from the half-triangular outlier component of the mixture model. The RT with a given $\widehat{CDF}$ value within the valid RT reference distribution is the corresponding numerical value of the outlier with that $\widehat{CDF}$ value. For example, for the UM outlier model and the low true proportion of outliers, the fitted mixture model includes outliers with a half-triangular distribution of $\widehat{CDF}$ values ranging from 0.971–1. The median of this distribution can be computed to be a $\widehat{CDF}$ of 0.9915.

What does this mean in terms of RTs? To have a concrete example, assume arbitrarily that the ex-Gaussian (*µ* = 300, *σ* = 30, *τ* = 100) distribution is chosen as the reference distribution of valid RTs. The mean and standard deviation of this distribution can be computed to be 400 ms and 104 ms, respectively. Relative to this reference distribution, the median outlier RT must be the RT with the CDF value of 0.9915—the previously computed median outlier $\widehat{CDF}$. Computations with this reference distribution show that this median outlier is an RT of 781 ms—a value which is approximately 3.8 standard deviations above the reference distribution’s mean. Thus, the mixture model provides not only an estimate of the proportion of outliers but also an estimate of the actual numerical values of those outliers relative to any given assumed reference distribution of valid RTs. Of course, these numerical values (e.g., 781, 3.8) are specific to this particular assumed reference distribution rather than values characteristic of RT tasks in general, but they illustrate how corresponding values could be obtained for a reference distribution regarded as typical for any particular task.

**Fig. 10:**
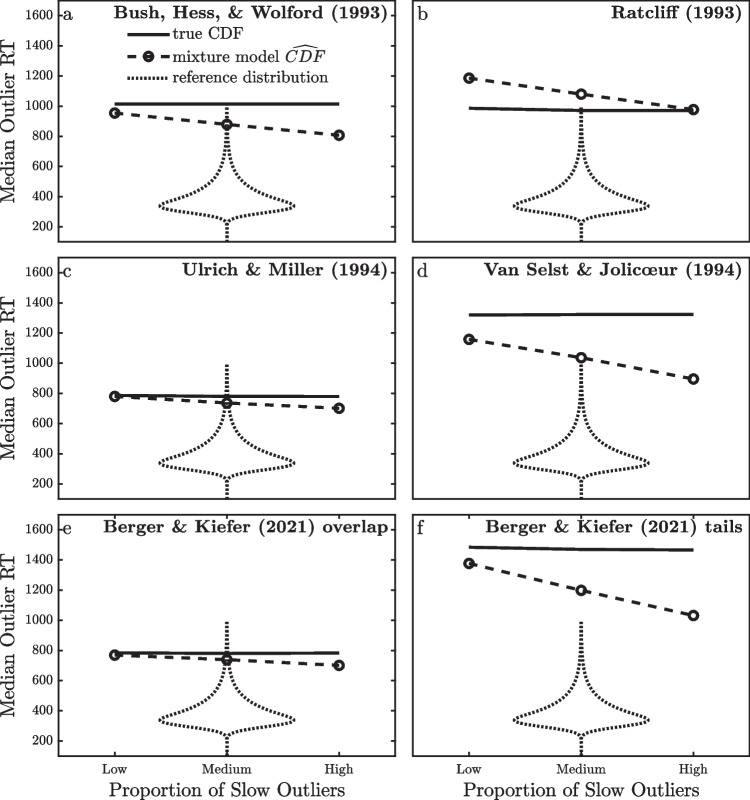
Median RTs of outliers relative to the ex-Gaussian (µ = 300, σ = 30, τ = 100) reference distribution, as computed from the true CDFs of the outliers and from the $\widehat{{\text{CDF}}}$ values estimated from the mixture model. The reference distribution of valid RTs shown as a violin plot was the same in all conditions

Figure 10 illustrates the accuracy of this procedure for estimating median outlier RTs using the simulated RTs from the models with known outliers in Fig. 1 and the ex-Gaussian (*µ* = 300, *σ* = 30, *τ* = 100) reference distribution shown for comparison in the violin plots. For each model and proportion of outliers, I first computed the median outlier $\widehat{CDF}$ within the half-triangular outlier distribution from the fitted mixture model, as explained above. The value of RT with that CDF value within the reference distribution is then the mixture model’s estimate of the median outlier RT relative to the reference distribution, and these values are plotted with the dashed lines. For comparison, the true CDF value of each simulated outlier RT was computed relative to each participant’s true valid RT distribution, and the median of these CDF values was determined. The value of the reference distribution with this median true CDF value, shown as the solid line, would be a better estimate of the median outlier RT, but it cannot be computed in practice because researchers can only compute estimated $\widehat{CDF}$ values—not true CDF values. Note that the true CDFs of the outlier RTs and their corresponding median in the reference distribution do not depend on the proportion of slow outliers. This proportion influences the estimation process through its impact on parameter estimation (e.g., Table 5), but it does not influence the true values of the outlier RTs or their true CDFs within the valid RT distributions.

The results shown in Fig. 10 suggest that the mixture model provides a reasonable estimate of the median outlier RT relative to the reference distribution. The medians estimated from the mixture model are not exactly equal to those computed from the true CDFs, of course, due to the estimation bias and random error inherent in that model. The estimated medians are generally similar to the true medians, though, at least when compared to the range of values in the reference distribution shown by the violin plots. Looking across the different panels of Fig. 10, it is clear that the mixture model generally reproduces the differences among models in the sizes of the outliers relative to the valid RTs. The mixture model’s predicted median outlier RTs are relatively fast (i.e., approximately 800 ms; Fig. 10c and e) for the outlier models whose outliers are only slightly slower than the slowest valid RTs (Fig. 1c and e), whereas the mixture model’s predicted median outlier RTs are distinctly slower (i.e., approximately 1000 ms or more; Fig. 10a, b, d, and f) for the outlier models whose slow outliers are more extreme (Fig. 1a, b, d, and f). The mixture model’s predicted median outlier RTs are generally less extreme than they should be, and this underestimation increases when there are more slow outliers. This pattern of underestimation is presumably a further consequence of the bias in *τ* estimation, which makes the estimated $\widehat{CDF}$ values less extreme than their true values. Interestingly, the RAT model is an exception to the general pattern of underestimation, as can be seen in Fig. 10b. In this case the mixture model’s estimate of the median outlier RT tends to be somewhat larger than the true value, unlike the estimates for the other outlier models. The explanation for this difference is not clear, but it is not surprising that the mixture approach works differently for this outlier model given that its outliers are qualitatively different than those of the other models and that the approach so badly overestimates this model’s minimum outlier $\widehat{CDF}$ , *ω*, in the first place (see Table 1).

It is even more interesting to see what this type of analysis suggests about the latencies of outliers in real RT data. Table 3 shows the median outlier RTs estimated as described above using $\widehat{CDF}$ values from the four real lexical decision task datasets, again using the ex-Gaussian (*µ* = 300, *σ* = 30, *τ* = 100) as the reference distribution. The results look reasonable for all of the datasets except FLP. The absolute times are plausible, with median outlier RTs approximately 4–6 standard deviations of RT above the mean of the valid RT distribution. In addition, the median outlier RTs are consistent across the four conditions within each experiment, as would be expected if the outliers were randomly distributed across conditions. The fact that the outliers were faster in the DLP dataset than in the SPP and ELP datasets could be due to the use of the shorter 2 s RT interval in the former study, as opposed to the 3 s and 4 s intervals in the latter studies. However, the median outlier RTs do not seem plausible in the FLP dataset—particularly for the nonword conditions—because they are not nearly slow enough. This is not unexpected given the weak evidence of slow outlier spikes in this experiment (Fig. 8) and the small *ω* estimates shown in Table 2, and it provides a further indication that slow outliers were rare or non-existent in this study. Again, if there is little or no evidence that slow outliers are present in the data, then there is little or no hope of estimating the minimum or median of the slow outlier latencies.

**Table 3:** Median outlier reaction times relative to the ex-Gaussian (µ = 300, σ = 30, τ = 100) reference distribution estimated via the mixture model fits shown in Table 2

	Word		Nonword	
Dataset	Rep.	Alt.	Rep.	Alt.
SPP	1079	1036	1139	1049
DLP	884	885	896	848
ELP	1050	978	1051	1032
FLP	717	724	494	530

Rep. = repetition. Alt. = alternation

## Robustness of the method

Although the new CDF pooling method appears to work quite well with the lexical decision task mega-study datasets examined here, it is reasonable to question how well it would work with other datasets^6^. For one thing, these datasets had large numbers of trials per participant in each condition, and it seems worthwhile to investigate how well the technique would work with fewer trials. Likewise, these datasets had relatively low proportions of outliers—seemingly only slow ones—and it is possible that the method would break down with larger proportions of outliers, outliers at both extremes, or both. This section reports separate sets of simulations investigating these two issues. Both sets of simulations used large numbers of simulated participants, so the results illustrate the long-term biases that can be expected to emerge when the method is used with fewer trials or more outliers.

### Number of trials

To investigate the number of trials per participant and condition needed for the method to work, I used the real RTs for the word, repetition condition of the ELP dataset (Fig. 7a), selecting this dataset because of its large RT cut-off (4 s) as well as its large numbers of participants and trials. In different simulations, I applied the method to randomly selected subsets of *N* = 25, 50, 75, 100, 125, or 150 trials from this condition for each participant. That is, the ex-Gaussian was fit separately to the RTs in each random subset of N trials, and the $\widehat{CDF}$ values of the RTs in that subset were computed relative to its fitted ex-Gaussian. For each N, this process was repeated enough times to get 3000 $\widehat{CDF}$ values for each of the 791 participants so that there would be a large number of $\widehat{CDF}$ values for that N, and finally all of the $\widehat{CDF}$ values were pooled together into a histogram for that N.

**Fig. 11:**
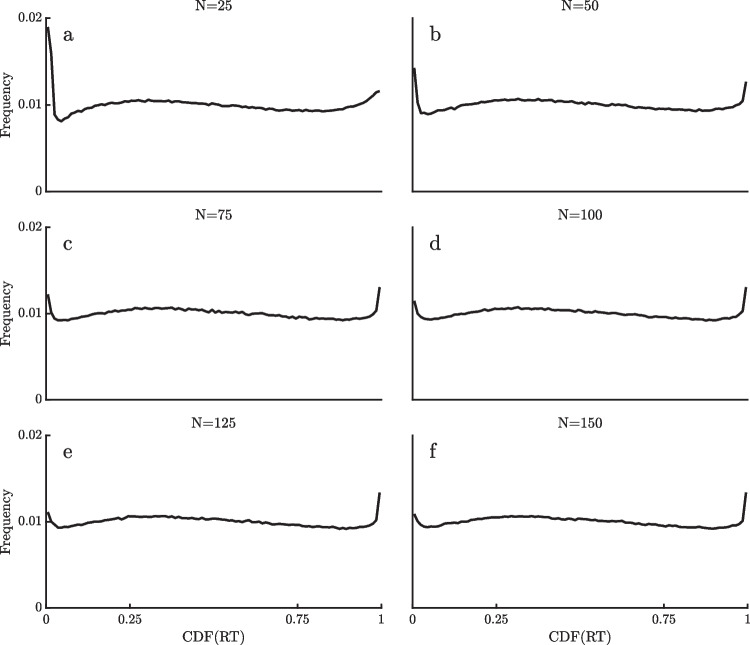
Histogram of pooled estimated cumulative distribution function ($\widehat{{\text{CDF}}}$) values of RTs for random subsets of different numbers of trials for the word, repetition condition of the English Lexicon Project dataset

The results of these simulations are shown in Fig. 11. To the extent that the method still works properly with a given sample size of N trials, the resulting histograms should show approximately the same high-end spikes as the original histograms. Not surprisingly, the method does break down with smaller numbers of trials. In particular, smaller N’s result in diminished high-end spikes and also produce low-end spikes that would suggest the presence of fast outliers, even though there was no evidence of fast outliers when all RTs were analyzed. These simulations suggest that at least 75 trials would be needed per participant in each condition for the method to be at all usable, but more trials would obviously be better. Inaccuracies in the estimation of the ex-Gaussian parameters are presumably responsible for the distortion of the pooled $\widehat{CDF}$ histograms with smaller Ns. Table 4 shows the means and standard deviations of the parameter estimates obtained with each of the different Ns, and parameter estimates appear to be less biased and more stable with larger Ns, as expected.

**Table 4:** Means and standard deviations (in parentheses) of the estimates of the parameters µ, σ, and τ of the ex-Gaussian distributions of reaction times as a function of the number of randomly selected trials included in the analysis

N	*µ*	σ	*τ*
25	516.02 (116.53)	58.565 (75.78)	279.73 (165.63)
50	502.29 (97.01)	60.293 (57.72)	293.48 (158.36)
75	499.11 (91.43)	61.965 (53.03)	296.51 (154.26)
100	498.62 (89.90)	63.967 (53.05)	296.98 (153.30)
125	497.79 (89.43)	64.335 (50.50)	297.93 (151.52)
150	497.70 (88.95)	64.745 (47.57)	298.08 (150.73)

### Proportion of outliers

An additional set of simulations looked at how the CDF pooling method fares with increases in the proportion of fast outliers, slow outliers, or both. The potential problem is that larger proportions of outliers could distort the estimates of the valid RT distribution’s ex-Gaussian parameters so seriously that the pooled $\widehat{CDF}$ values would no longer produce the discernible low- and high-end spikes that tend to indicate the presence of outliers.

To allow precise control over the true proportion of outliers, in these simulations the valid RTs were generated from true underlying ex-Gaussian distributions, whereas the fast and slow outliers were generated in accordance with a mixture model using half-triangular CDF distributions like the one shown in Fig. 9. To ensure realistic variation across simulated participants, the parameter values of the valid RT ex-Gaussians were sampled from the individual-participant parameter values estimated for the word, repetition condition from the full ELP dataset. The probabilities of fast and slow outliers, *p*_*o*_, were varied across simulations. In some simulations there were only fast outliers, in others there were only slow outliers, and in others there were both fast and slow outliers, each with probability *p*_*o*_, so that the probability of a valid RT was 1 *−* 2 *× p*_*o*_ in these latter simulations. Each slow outlier was an RT whose CDF within the valid ex-Gaussian came from a half-triangular distribution with *ω* = 0*.*99, and each fast outlier was an RT whose CDF came from a mirror-reflected half-triangular distribution in the range of 0–0.01 (i.e., maximum density at CDF equal to 0, decreasing linearly to 0.01).

**Fig. 12:**
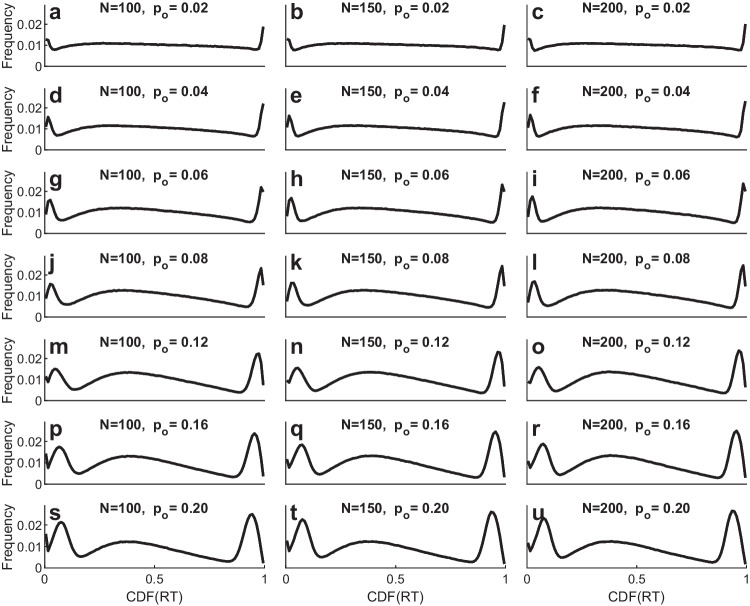
Histogram of pooled estimated cumulative distribution function ($\widehat{{\text{CDF}}}$) values of RTs for random samples of different numbers of ex-Gaussian RTs using parameters estimated from the word, repetition condition of the English Lexicon Project dataset, with different probabilities (p_o_) of fast and slow outliers

Figure 12 shows the results of the simulations in which there were both fast and slow outliers. The frequency distributions of $\widehat{CDF}$ values are reasonably uniform apart from the expected low-end and high-end spikes as long as the outlier probability is less than approximately 0.06, especially with 150–200 trials. The frequency distributions are clearly distorted, however, when the proportions of outliers are larger. Both the low-end and high-end spikes seem to be shifted slightly inwards from the most extreme boundaries of zero and 1. For example, the largest spike associated with the high-end outliers moves down into the 0.97–0.99 bins of $\widehat{CDF}$ values rather than being concentrated in the top 0.99–1 bin. The pattern is accompanied by under-representation of $\widehat{CDF}$ values in the bins from approximately 0.50 to 0.97. Such a distinctive pattern of departures from the expected uniform $\widehat{CDF}$ distribution may thus be suggestive of a relatively large proportions of outliers in an RT dataset.

The pooled $\widehat{CDF}$ values that emerged from the simulations with just one type of outlier—either fast or slow—were quite similar to the ones shown in Fig. 12 at the end with the outliers, and they were reasonably flat otherwise. Thus, it appears that fast and slow outliers have relatively independent effects on the pooled $\widehat{CDF}$ values, at least with proportions and latencies of outliers comparable to those used in these simulations.

**Fig. 13:**
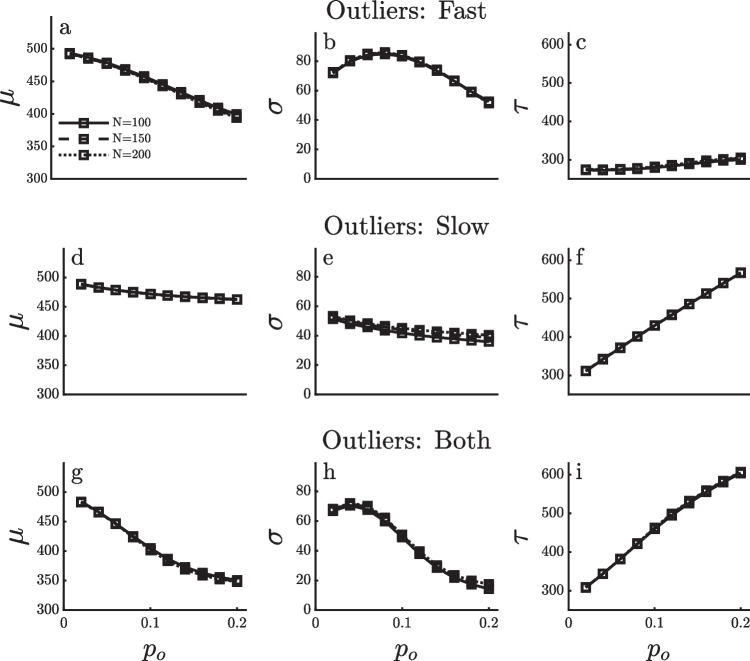
Average estimated values of ex-Gaussian distribution parameters obtained from samples of N trials with different probabilities (p_o_) of fast and slow outliers. The true average parameter values were µ = 494.9, σ = 59.0, and τ = 280.6

Figure 13 shows how the presence of outliers affected the average ex-Gaussian parameter estimates used in computing the pooled $\widehat{CDF}$ values for these simulations. Clearly, the strongest effect of fast outliers was to bias the estimates of *µ* toward smaller values, whereas the strongest effect of slow outliers was to bias the estimates of *τ* toward larger values. Each of these biases would make the outliers appear to have less extreme $\widehat{CDF}$ values, which may account for the inward shifts of the fast and slow outlier spikes away from the boundaries of zero and 1.

## General discussion

### CDF pooling method

The CDF pooling method presented here provides a bottom-up tool for obtaining an empirical description of outliers in real RT distributions, thereby enabling the study of the characteristics of these outliers. In contrast, most previous attempts to describe outliers have been top-down in the sense of assuming a particular classification criterion a priori (e.g., *>* 2*.*5 SDs above the mean) and then comparing trials that were versus were not classified as outliers (e.g., Berger & Kiefer, 2023; Miller, 2023). Although such comparisons can reveal differences between relatively fast and slow RTs, they are not necessarily indicative of the properties of outliers per se, because valid RTs may themselves be slow.

An important strength of the new method is that it allows researchers to summarize overall RT distributions using virtually unlimited numbers of trials, because there is no limit on the number of participants whose $\widehat{CDF}$ values can be pooled together within a distribution. Thus, this method can provide arbitrarily high resolution, which is essential when trying to get a picture of rare events such as RT outliers. In fact, because $\widehat{CDF}$ distributions should in principle be uniform for all conditions as well as for all participants, it might also be possible to pool them across conditions and even across experiments in order to get a picture of the outliers within a wider domain. In contrast, the most common previous method of RT distributional analysis—known as “Vincentizing”—is to compute the quantiles of the observed RTs for each participant and then to average these quantiles (Ratcliff, 1979; Vincent, 1912) or some transformation of them (Cousineau et al., 2016) across participants. Such quantile averaging has lower resolution than CDF pooling, because the number of quantiles cannot exceed the number of RTs per participant. With Vincentizing, researchers would need thousands of trials *per participant in each condition* to study rare outlier RTs, and even with that many trials there would be distortions due to averaging the outlier RTs of some participants with the valid RTs of other participants at a given high quantile (e.g., participants with 1% versus 2% outliers).

In principle, the new CDF pooling method can be used with any distribution—not just the ex-Gaussian. The major limitation of the pooling method is that it requires commitment to a specific RT distribution for computing the $\widehat{CDF}$ values of the individual RTs of a given participant in a given condition [e.g., ex-Gaussian (*µ* = 305, *σ* = 28, and *τ* = 174)]. Thus, researchers must select a particular distribution family and have enough trials per participant in each condition to get reasonably accurate estimates of its parameters. Fortunately, the observed RT distributions themselves can be checked when selecting the appropriate distribution family (see Appendix 2), which provides empirical guidance. Moreover, the method provides an internal check: the final distribution of pooled $\widehat{CDF}$ values should be approximately flat, apart from outliers at one or both ends, if an appropriate distribution family has been chosen and its parameter estimates are sufficiently accurate. Likewise, unreasonable values of the estimated mixture model parameters (e.g., *ω <* 0*.*95) would indicate either that there were no outliers or that the model was not appropriate for a given dataset, as was the case with the FLP dataset examined here.

It is not possible to be completely certain about the accuracy of the new method’s performance in real datasets without having a pre-existing gold standard indicator of which RTs are outliers, but unfortunately no such standard is available. In the absence of such a standard, the results of simulations using the previously suggested outlier models (Fig. 1) and using distributions with known percentages of outliers (Fig. 12) provide encouragement that the method can reveal fundamental information about outliers when there are enough trials per condition and participant.

### Outliers in lexical decision tasks

Because the new method was used here with a restricted set of real datasets (i.e., lexical decision tasks with limited response intervals and normal adult participant populations), the information about outliers obtained from the current analyses is—strictly speaking—limited to this task and population type. Nonetheless, because these tasks and populations are representative of many published RT experiments, it seems appropriate to regard the present conclusions about outliers in these tasks as a plausible tentative description of outliers in a variety of RT studies. Further use of the method within a wide variety of studies will be extremely useful for increasing knowledge about the frequency and characteristics of RT outliers more generally.

With respect to the real lexical decision task datasets examined in the present case study, the present analyses using the CDF pooling method suggest several conclusions about the shapes of RT distributions and the characteristics of the outliers they contain. First, outliers appear to be quite rare in these tasks. Apart from the obviously anomalous RTs less than 150 ms, there was no evidence of any fast outliers. Slow outliers were present in all conditions for three of the four datasets and in two conditions for the fourth, but these were also quite rare—probably less than 0.5%—supporting the conclusions reached through different types of analyses of the same datasets by Miller (2023).

Second, the slow outliers seemed to be in the range of approximately 4–6 standard deviations of RT above the mean of the valid RT distribution. Although the slow outlier RTs were necessarily at the upper ends of the distributions of valid RTs, there did seem to be some overlap between the valid RTs and outliers, presumably because of the long positive tail of the valid RT distribution. Based on the values of *ω* from the mixture model summarized in Table 2, the distribution of outlier RTs often overlapped with the top 1–1.5% of the valid RT distribution, except for the FLP dataset for which this parameter has little meaning due to the absence of outliers in the first place.

Third, the ex-Gaussian distribution provided a good description of individual RT distributions in all of these datasets. This finding is consistent with many previous reports of good fits for the ex-Gaussian (e.g., Balota & Yap, 2011; Hohle, 1965; Luce, 1986), and it extends those reports because the ex-Gaussian was pitted against an unusually large number of competing distributions. The near uniformity of the $\widehat{CDF}$ values obtained from fitted ex-Gaussians and pooled across huge numbers of trials (e.g., Fig. 5) also provides further evidence that this distributional model is appropriate. The presence of high-end spikes in these pooled $\widehat{CDF}$ distributions also suggests that the ex-Gaussian parameter estimation is robust enough to tolerate occasional outliers, although the simulations with added outliers show that the pooled CDF distributions may take on a different shape when the proportion of outliers exceeds 5–6% (Fig. 12).

### Implications for outlier exclusion procedures

The overlap between the distributions of valid and outlier RTs suggested by the present analyses makes it very difficult to come up with a method of classifying valid versus outlier trials when RT is the only variable used for classification. Given the overlap of valid RTs and outliers, any given cut-off will either reject some valid RTs as outliers, accept some outliers as valid RTs, or both. On the other hand, the overlap suggests that outliers in RT tasks may not generally be atypical enough to seriously distort the summary results (e.g., sample mean), especially considering that they seem to be rare.

The present results provide some guidance for researchers wanting to use existing outlier exclusion procedures. As discussed by Miller (2023), almost all existing outlier exclusion procedures involve an experimenter-determined cut-off defining how extreme an RT must be in order to be classified as an outlier (e.g., *>* 2*.*5 SDs above the sample mean). The fact that there seem to be few slow outliers in the present datasets suggest that relatively extreme cut-offs should be chosen. With the ex-Gaussian (*µ* = 300, *σ* = 30, *τ* = 100) distribution, for example, the commonly-used 2.5 SD cut-off would classify approximately 3% of RTs as outliers; a rarely used 3.5 SD cut-off would be needed to reduce that percentage to the evidently more appropriate value of 1%. The cost of identifying valid RTs as outliers, of course, is loss of power, not only because of the smaller number of trials included in the analysis but also because slow valid RTs may be particularly informative when the effects of interest are largest for the slowest responses.

Naturally, information about the empirical characteristics of outlier RTs can also inform the search for better outlier exclusion procedures. In particular, when simulations are conducted to compare different procedures, the simulation conditions should reflect empirically realistic conditions. The present results strongly suggest limits on those conditions. For example, in hindsight, the values of 5–10% slow outliers used in most simulations (e.g., Berger & Kiefer, 2021; Ratcliff, 1993; Ulrich & Miller, 1994; Van Selst & Jolicœur, 1994) seem unrealistically large, and the value of 1% slow outliers used by Bush et al. (1993) seems more appropriate. Likewise, the present results also suggest that the simulated slow outlier RTs should not be made too extreme relative to the valid RTs, with an outlier median only approximately 4–6 standard deviations of RT above the mean of the valid RT distribution.

More speculatively, the method of CDF pooling could be extended to form the basis of a new RT outlier exclusion procedure. Researchers could first examine the observed RTs from all participants, select an appropriate distribution family, and then use the mixture model to estimate the minimum $\widehat{CDF}$ values of outliers, *ω*. The $\widehat{CDF}$ of each RT from each participant could then be computed, and any RT with $\widehat{CDF}$(*RT*) *> ω* could be classified as an outlier. While this procedure has some intuitive plausibility based on the current analyses, empirical examination would be required to evaluate its performance with actual datasets (cf. Miller, 2023).

## Conclusions

The novel CDF pooling method appears to be a promising approach for obtaining the high-resolution information about RT distributions needed to study the distributions’ tails. It performs well with several previously suggested models of what real outliers might look like, producing useful approximations to the known probabilities and latencies of outliers within those models. When applied to real data, the method produces reasonable results that pass a number of internal consistency checks. Thus, the method provides a plausible procedure for learning about outliers in real datasets. The information gained would be useful for assessing the likely effects of outliers on different methods of RT analyses, developing new classification criteria for outlier exclusion, and identifying factors that contribute to the occurrence of outliers in the first place.

## Figures and Tables

**Fig. 14:**
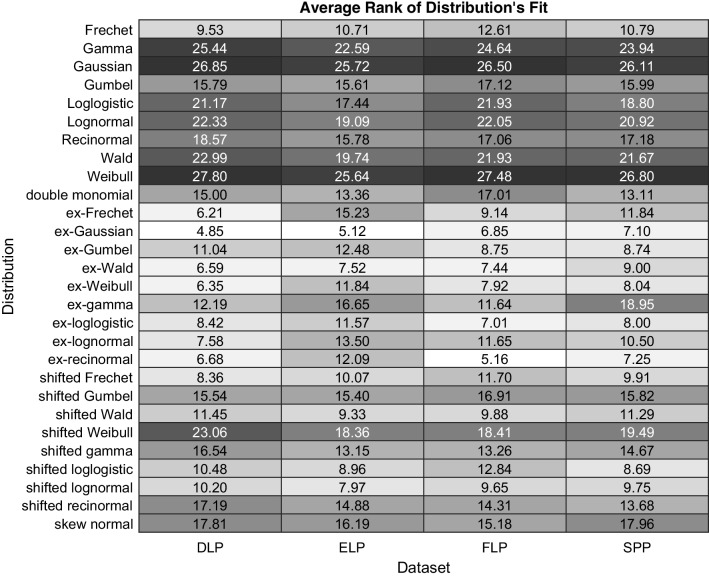
Heat map showing the average rank of each of the candidate reaction time (RT) distributions shown in Table 6, with lower average ranks indicating better fits

## Data Availability

R routines for estimating parameters of the ex-Gaussian distribution and computing the to-be-pooled CDF values are available at https://github.com/milleratotago/CDFpooling.

## Appendix 1

### Simulations of outlier models

This appendix presents further information about the simulation methods and results obtained with the outlier models shown in Fig. 1.

#### Simulation method

For all models, valid RTs were randomly sampled from ex-Gaussian distributions with parameters of *µ*, *σ*, and *τ*. Following Berger and Kiefer (2021), random participant-to-participant variation was implemented by sampling each participant’s values of these three parameters from uniform distributions (*µ*: 250–500 ms; *σ*: 20–50 ms; *τ*: 150–200 ms). The proportion of true outliers also varied randomly across participants. In simulations with a low, medium, or high proportion of outliers, each participant’s probability of a slow outlier was randomly selected from a uniform distribution ranging between 0–0.02, 0–0.05, or 0–0.1, respectively.

For the models depicted in Fig. 1a and b, the distribution of slow outlier latencies was determined as specified in the original theoretical model (e.g., a valid RT plus a uniform increment of 0–2000 ms for the model of Ratcliff, 1993). For the outlier model of Ulrich and Miller (1994), the distribution of slow outliers was an ex-Gaussian whose parameters depended on the parameters of the participant’s valid RT distribution in the same way as in the overlap model of Berger and Kiefer (2021), as described in the main text. For the model of Van Selst and Jolicœur (1994), the distribution of slow outliers was an ex-Gaussian whose *µ* was 9.3 valid distribution *σ*s larger than the valid distribution’s *µ*, as in the original simulations carried out with that model by Van Selst and Jolicœur (1994). For the BK_overlap_ method depicted in Fig. 1e, each participant’s outliers came from specific ex-Gaussian distributions whose parameters depended on the randomly selected parameters of that participant’s valid RT distribution as specified by Berger and Kiefer (2021). For the BK_tails_ method shown in Fig. 1f, again following Berger and Kiefer (2021), the outliers were defined relative to the observed minimum and maximum RTs in any given *sample* of valid trials, so an outlier in one sample was always more extreme than any valid RT in that sample, though it could be less extreme than a valid RT in some other sample. Using these procedures, a sample of 200 RTs was generated for each participant.

#### Analysis and results

For all models, the CDF value of each randomly generated RT—valid or outlier—was determined relative to the true ex-Gaussian distribution of valid RTs for the simulated participant (i.e., with that participant’s true values of *µ*, *σ*, and *τ*). These CDF values were pooled across simulated participants, and the dots in Fig. 4 show the relative frequencies of these different CDF values.

In addition, each sample of 200 RTs for each simulated participant was used to obtain maximum likelihood estimates of the best-fitting ex-Gaussian parameters (i.e., $\widehat{\mu }$, $\widehat{\sigma }$, and $\widehat{\tau }$). Then, the estimated $\widehat{CDF}$ value of each RT in the sample was determined relative to the ex-Gaussian distribution with these estimated parameters. These $\widehat{CDF}$ values were also pooled across simulated participants, and the histogram bars in Fig. 4 show the relative frequencies of these different $\widehat{CDF}$ values.

As noted in the main text, the differences between the frequencies of CDF and $\widehat{CDF}$ values are caused in large part by biases in the parameter estimates $\widehat{\mu }$, $\widehat{\sigma }$, and $\widehat{\tau }$ resulting from the presence of the outliers. Table 5 summarizes these biases. Each bias was computed as the mean across simulated participants of the estimated parameter value minus the true parameter value, so positive biases indicate that the parameters estimated from the RT samples were larger than the true parameters of the underlying ex-Gaussian distributions of valid RTs.

The results in Table 5 mainly show that the estimated values $\widehat{\tau }$ are larger than the true *τ* values and that this overestimation increases with larger proportions of outliers. This makes sense, since slow outliers make it look as if the RT distribution has a longer tail at the high end, and the *τ* parameter increases with the length of this tail. There are also smaller biases in the estimates of *µ* and *σ*, and these values tend to be underestimated relative to the true values. These biases can be understood as a secondary consequence of the overestimation of *τ*. When too much of the observed RT mean and variance are allocated to *τ*, there is correspondingly not enough left over to be allocated to *µ* and *σ*.

**Table 5:** Bias in milliseconds of the estimates of the parameters µ, σ, and τ of the ex-Gaussian distributions of valid reaction times (RTs) as a function of the outlier generation model and the simulated proportion of outliers (low, medium, high)

Outlier	*µ*			*σ*			*τ*		
Model	Low	Medium	High	Low	Medium	High	Low	Medium	High
BHW	-3	-6	-10	-3	-4	-6	14	33	64
RAT	-2	-6	-10	-2	-4	-6	11	31	59
UM	-1	-4	-6	-2	-3	-4	8	22	39
VSJ	-4	-9	-15	-3	-6	-9	19	50	102
BK_overlap_	-1	-4	-7	-2	-3	-5	8	22	43
BK_tails_	-5	-11	-17	-4	-7	-9	25	58	113

For each simulated participant, bias was computed as the estimated parameter value minus the true parameter value, and these biases were averaged across simulated participants. Outlier models: BHW = Bush et al. (1993); RAT = Ratcliff (1993); UM = Ulrich and Miller (1994); VSJ = Van Selst and Jolicœur (1994); BK_overlap_ = overlap model of Berger and Kiefer (2021); BK_tails_ = tails model of Berger and Kiefer (2021)

## Appendix 2

### Selecting the reaction time distribution family

As was illustrated in the section “Pooling CDF values,” the proposed method of constructing a pooled distribution of $\widehat{CDF}$ values requires the specification of a distribution family that is a good approximation of the true underlying RT distributions. This appendix describes the different RT distribution families that were considered for the present analyses and shows the results supporting the choice of the ex-Gaussian family for the present datasets.

The choice of an RT distribution family is complicated, partly because of the large number of different distribution families that have been suggested as possible candidate models of RTs (e.g., Luce, 1986). There have been few previous attempts to compare the fits of alternative distribution families to empirical datasets, and these have compared only a few of the suggested families at a time (e.g., Palmer et al., 2011). Furthermore, the proposed distribution families are all somewhat similar, including long tails at the high end of the RT distribution, so it is difficult to tell them apart. Fortunately, for the present purposes, it is probably not crucial that the chosen distribution be exactly correct; it only needs to be a good enough approximation to produce nearly correct $\widehat{CDF}$ values. Thus, the goal of the present analysis was to select the best available distribution family as a model for RTs, without making any claims that this is necessarily the “true” distribution.

### Candidate distribution families

In the RT literature, the ex-Gaussian is by far the most-often considered descriptive distributional family (e.g., Burbeck & Luce, 1982; Hohle, 1965). An informal survey of the literature shows than many other distribution families have also been considered, however, including the lognormal (e.g., Ratcliff & Murdock, 1976; Schlosberg & Heineman, 1950; Storms & Delbeke, 1992; Ulrich & Miller, 1993), gamma (e.g., Lo & Andrews, 2015; McGill & Gibbon, 1965; Palmer et al., 2011), Weibull (e.g., Logan, 1988; Palmer et al., 2011), Wald (e.g., Cousineau et al., 2004; Dolan et al., 2002; Heathcote, 2004), shifted Wald (e.g., Anders et al., 2016; Castro et al., 2019), ex-Wald (e.g., Heathcote, 2004; Holden & Rajaraman, 2012; Palmer et al., 2011; Rieger & Miller, 2020; Schwarz, 2001), recinormal (e.g., Carpenter & Williams, 1995; Lo & Andrews, 2015), loglogistic (e.g., Cousineau et al., 2016), Gumbel (e.g., Cousineau et al., 2016), Frechet (e.g., Luce, 1986), and double monomial (e.g., Luce, 1986; Snodgrass et al., 1967). All of these distributions were considered here. To search even more thoroughly for the best-fitting distribution, I also considered a number of other distributions that could be derived from these distributions by adding a constant shift or an exponentially-distributed shift.

Table 6 lists all 28 of the distribution families considered in the present work. Nine families have two parameters, and all are skewed except for the Gaussian, which was only included for completeness. An additional version of each of these nine families was constructed by including a third parameter reflecting a positive shift added to scores in the distribution, except for the Gaussian for which a shift is redundant with a change in *µ*. Furthermore, for each of these families, a second additional version of the family was formed as the convolution of the family with an exponential distribution—the mean of the exponential thus being a third parameter. Two of these are already included in the list of previously considered distributions, of course—namely, the ex-Gaussian and ex-Wald. Finally, there were two inherently three-parameter families as shown at the bottom of the table. For each of the 28 families in Table 6, MATLAB routines were developed to estimate distribution parameters using maximum likelihood (cf., Cousineau et al., 2004), and these routines are freely available as part of an updated MATLAB Cupid package (Miller, 1998) available at https://github.com/milleratotago/Cupid.

Separately for RTs of each of the participants in each dataset and each of the four experimental conditions being distinguished (i.e., word/nonword x response repetition/alternation), parameters were estimated by maximum likelihood for each of the distributions in Table 6,^7^. Then, for each combination of participant and condition, the different distributions were ranked from best to worst (i.e., 1–28) in accordance with the maximum likelihood value estimated for that distribution. Finally, for each dataset, the average rank was computed for each of the distributions in Table 6, averaging across the participants and the four experimental conditions^8^.

Figure 14 is a heat map showing the average ranking of each distribution’s fits to the individual participant and condition RT distributions for each of the datasets, with lower numbers indicating better fits. The ex-Gaussian distribution provides the lowest average rank for three of the four datasets and the second-lowest rank for the fourth (i.e., French Lexicon Project), so it seemed appropriate to choose this as the most plausible descriptive family for the observed RT distributions. Notably, the similar ex-Wald distribution (Schwarz, 2001, 2002) also does reasonably well, whereas several other distributions that have been suggested as RT models fare quite badly in this comparison (e.g., recinormal, loglogistic, shifted Weibull). It is particularly interesting that the ex-Gaussian fits best in these lexical decision task datasets, because part of the RT variance was no doubt due to differences among items (i.e., different words or different nonwords). Thus, the theoretical distributions of RTs to individual words might not be ex-Gaussian, with the ex-Gaussian shape only emerging after pooling RTs across different words.

**Table 6:** The 28 probability distributions considered as candidate models of within-participant, within-condition reaction time distributions

Distribution Family and Version		
Distribution	Shifted	Summed with exponential
*two-parameter families:*		
Frechet	shifted Frechet	ex-Frechet
gamma	shifted gamma	ex-gamma
Gaussian		ex-Gaussian
Gumbel	shifted Gumbel	ex-Gumbel
loglogistic	shifted loglogistic	ex-loglogistic
lognormal	shifted lognormal	ex-lognormal
recinormal	shifted recinormal	ex-recinormal
Wald	shifted Wald	ex-Wald
Weibull	shifted Weibull	ex-Weibull
*three-parameter families:*		
double monomial		
skew normal		

The shifted Frechet and shifted Weibull families considered here are special cases of the standard three-parameter versions of the Frechet and Weibull distributions, with the additional constraint being that the shift parameter was constrained to be positive

## Footnotes

1 Bush et al. (1993) were only interested in having a distribution with an appropriate skew, so they simulated valid RTs as coming from chi-square distributions with means of 4–12 ms. To produce valid RTs in a realistic range for this illustration, I instead used a gamma distribution for the valid RTs with parameters adjusted to give realistic RTs.

2 For both the overlap and tails methods, the probabilities of outliers and the parameters of the distributions of valid RTs, fast outliers, and slow outliers varied randomly from participant to participant according to higher-level distributions (e.g., the ex-Gaussian *µ* parameter varied across participants according to a uniform distribution from 250–500 ms). Figure 1e and f depict distributions for a single participant with parameter values at the medians within each of their assumed higher-level distributions.

3 As is standard, “hats” are used in this article to indicate values that are estimates derived from sample data rather than true values.

4 In fact, most of the excluded RTs were less than 50 ms, and these are well outside the range of potentially valid observations in a choice RT task. Including the approximately 0.03% of trials in the 50–150 ms range had only miniscule effects on the analyses reported here. There were even smaller percentages of trials in the 50–150 ms range for two of the three additional datasets analyzed subsequently, but 0.34% of trials were in this range for the English Language Project dataset. Even for this dataset, however, there was virtually no effect of including these trials in the analyses.

5 Additional simulations were conducted with versions of the RAT model in which the outlier RTs were convolutions of the valid ex-Gaussian RT with a uniform delay ranging from 500–2000 ms or 1000–2000 ms, thus reducing the overlap of the valid RT and outlier distributions. The CDF pooling method was much more effective in detecting the proportions and minimum $\widehat{CDF}$ values of outliers in these simulations than in the original simulations using the 0–2000 ms delays used by Ratcliff (1993), highlighting the importance of reduced valid/outlier overlap for obtaining accurate results with the CDF pooling method and mixture model.

6 I thank Denis Cousineau for raising the issues addressed in this section.
